# Genome-wide transcriptional profiling provides clues to molecular mechanisms underlying cold tolerance in chickpea

**DOI:** 10.1038/s41598-023-33398-3

**Published:** 2023-04-18

**Authors:** Alireza Akbari, Ahmad Ismaili, Nazanin Amirbakhtiar, Masoumeh Pouresmael, Zahra-Sadat Shobbar

**Affiliations:** 1 Department of Plant Production and Genetic Engineering, Faculty of Agriculture, Lorestan University, Khorramabad, Iran; 2 Genetic Research Department, Seed and Plant Improvement Institute, Agricultural Research, Education and Extension Organization, Karaj, Iran; 3 Department of Systems Biology, Agricultural Biotechnology Research Institute of Iran (ABRII), Agricultural Research, Education and Extension Organization, Karaj, Iran

**Keywords:** Agricultural genetics, Gene expression, Gene expression analysis, RNA sequencing, Transcriptomics

## Abstract

Chickpea is an important food legume cultivated in several countries. A sudden drop in autumn temperature, freezing winter temperature, and late spring cold events result in significant losses in chickpea production. The current study used RNA sequencing of two cold tolerant (Saral) and sensitive (ILC533) Kabuli chickpea genotypes to identify cold tolerance-associated genes/pathways. A total of 200.85 million raw reads were acquired from the leaf samples by Illumina sequencing, and around 86% of the clean reads (199 million) were mapped to the chickpea reference genome. The results indicated that 3710 (1980 up- and 1730 down-regulated) and 3473 (1972 up- and 1501 down-regulated) genes were expressed differentially under cold stress in the tolerant and sensitive genotypes, respectively. According to the GO enrichment analysis of uniquely down-regulated genes under cold stress in ILC533, photosynthetic membrane, photosystem II, chloroplast part, and photosystem processes were enriched, revealing that the photosynthesis is severely sensitive to cold stress in this sensitive genotype. Many remarkable transcription factors (*CaDREB1E, CaMYB4*, *CaNAC47, CaTCP4*, and *CaWRKY33*), signaling/regulatory genes (*CaCDPK4*, *CaPP2C6, CaMKK2*, and *CaHSFA3*), and protective genes (*CaCOR47*, *CaLEA3*, and *CaGST*) were identified among the cold-responsive genes of the tolerant genotype. These findings would help improve cold tolerance across chickpea genotypes by molecular breeding or genetic engineering.

## Introduction

The third most significant pulse grown in the world is chickpea (*Cicer arietinum*)^1,2^. Cultivated chickpea, a diploid (2n = 2x = 16) plant with relatively small genome size, is an annual, self-pollinating crop^3,4^. Chickpea seeds are an excellent source of protein, essential amino acids, carbohydrates, starch, and fat^5^. Moreover, it has several advantages for agroecosystems through biological nitrogen fixation and soil fertility improvement^5,6^. Chickpea is widely cultivated in several parts of the world; in 2020, its production from an area of 14.84 million ha was estimated at 15.08 million tons globally. Generally, chickpeas are classified into two types, Kabuli and Desi. Kabuli seeds are typically large with a thin coat, mainly cream or beige in color. While the Desi type usually has small seeds with a wide range of diversity in testa color, including cream, yellow, brown, black, and green, as well as a thick coat^7^.

Abiotic stresses, including extreme temperatures^8–11^, salinity^12^ and drought^13^ are important environmental challenges for producing crops. Chickpea is classified as a chilling-susceptible species^14^. A sudden drop in autumn temperature, freezing winter temperature, and late spring cold events result in significant losses in chickpea production (about 40% overall reduction)^15^. Although all chickpea growth stages can be damaged by cold stress, the reproductive phase is the most sensitive stage^7^. Plants respond to cold stress by regulating the expression of stress-responsive genes, resulting in changes in several biochemical, physiological and molecular processes^10,16,17^. Identifying the genes related to cold stress response can prominently help the development of cold tolerance cultivars using molecular breeding and/or biotechnological approaches. A few studies have concentrated on detecting the cold tolerance-related genes in chickpeas^8,18,19^ but considered only one genotype and/or were restricted by sequence unavailability of the reference genome/transcriptome. Understanding the biology of tolerance mechanism to complex environmental stresses, including cold stress, needs high throughput genomics data.

The "omics" approaches have become an impartible part of scientific studies to determine plant responses to different stress conditions. The transcriptome could illustrate the functional part of the genome at each stage of plant growth. Transcriptomics discloses variations in the expression patterns of genes along with the regulatory mechanisms controlling differential gene expression. Therefore, it could be used as an efficient tool to precisely describe the mechanisms that lead to resistance or sensitivity^7^. The scientific collaboration of International Crops Research Institute for the Semi-Arid Tropics (ICRISAT) and other research organizations lead to sequence of the chickpea genome and the identification of over 28,000 genes and millions of genetic markers^20,21^.

Chickpea is traditionally planted in spring as a rainfed crop in Iran. High temperature and low precipitation in the crucial growth period result in terminal drought stress and low performance in plants. To overcome the mentioned problems, planting in autumn is suggested as a suitable agronomical approach; however, the lack of cold-tolerant chickpea cultivars is the limiting factor. Thus, it is necessary to develop cold-tolerant chickpea cultivars for cold regions of Iran. Discovering genes and mechanisms engaged in chickpea cold tolerance is important for developing cold tolerant cultivars. As an accurate technique to study the whole transcriptome, RNA-seq has been broadly utilized to examine cold stress response in plants^22,23^. Therefore, in the current research, two contrasting Kabuli chickpea genotypes (tolerant and sensitive) were subjected to deep transcriptome sequencing, and their expression profiles in response to cold stress were investigated. Comparing cold-responsive genes in the sensitive and cold tolerant genotypes led to identifying some promising candidate genes possibly involved in chickpeas' cold tolerance. Novel genes were also identified in the investigated genotypes. Furthermore, metabolic and biochemical pathways engaged in cold stress response were recognized by functional categorization of differentially expressed genes.

## Results

### Sequencing statistics and mapping results

A total of 200.85 million raw reads were acquired from all the samples by Illumina sequencing. Deleting adapters and low-quality reads caused 199 million clean reads which more than 88.70% of them had Phredlike quality scores at the Q30 level (Table S2). According to the results, on average, around 86% of the high-quality reads mapped to the chickpea reference genome, among which 80.38–81.38% in Saral and 80.36–82.21% in ILC533 were matched uniquely (Table 1).

**Table 1:** Summary of Illumina transcriptome reads mapped to the reference genome.

Reads mapping	Reads number (%)			
Sample	Saral N1	saralN2	Saral stress1	Saral stress2
Total reads	48,454,324	51,540,316	53,706,886	43,366,108
Total mapped reads	41,832,335 (86.33%)	44,397,736 (86.14%)	46,217,826 (86.06%)	37,052,553 (85.44%)
Unique match	39,434,666 (81.38%)	41,930,431 (81.35%)	43,679,714 (81.33%)	34,857,942 (80.38%)
Multi-position match	2,397,669 (4.95%)	2,467,305 (4.79%)	2,538,112 (4.73%)	2,194,611 (5.060%)
Total unmapped reads	6,621,989 (13.67%)	7,142,580 (13.86%)	7,489,060 (13.94%)	6,313,555 (14.56%)

Sample	ILC533 N1	ILC533N2	ILC533 stress1	ILC533 stress2
Total reads	49,205,474	48,070,120	53,223,910	54,140,088
Total mapped reads	42,279,998 (85.93%)	40,963,728 (85.22%)	46,372,063 (87.13%)	47,062,224 (86.92%)
Unique match	39,785,204 (80.86%)	38,629,667 (80.36%)	43,760,670 (82.22%)	44,380,164 (81.97%)
Multi-position match	2,494,794 (5.07%)	2,334,061 (4.86%)	2,611,393 (4.91%)	2,682,060 (4.95%)
Total unmapped reads	6,925,476 (14.07%)	7,106,392 (14.78%)	6,851,847 (12.87%)	7,077,864 (13.08%)

### Identification of cold responsive genes

**Figure 1:**
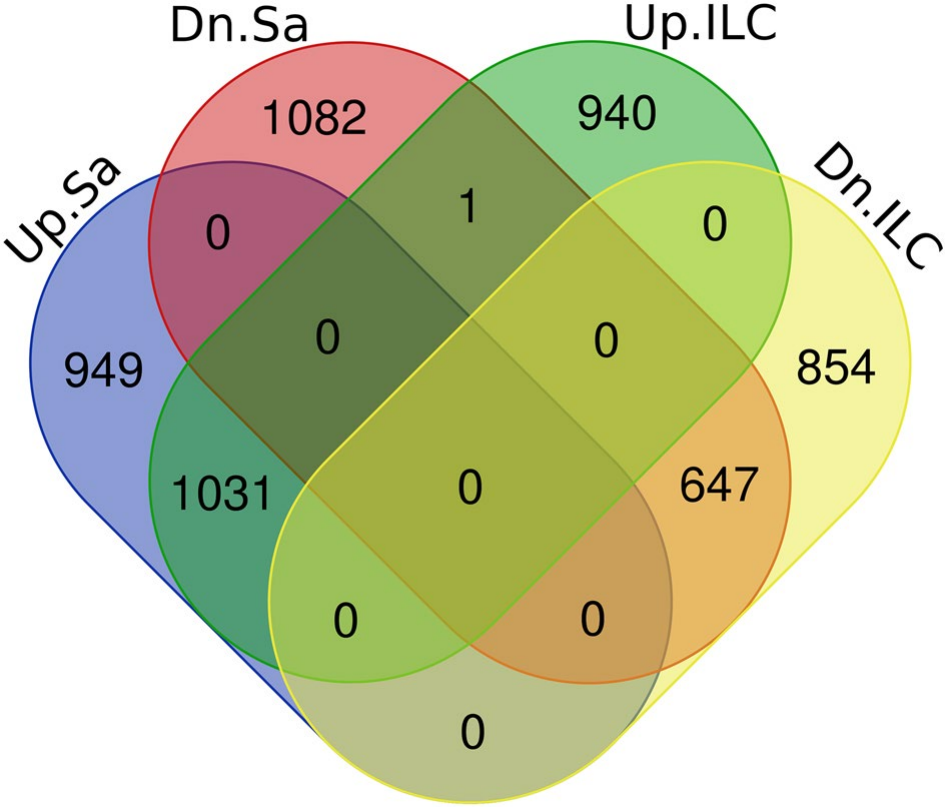
Venn diagram of differentially expressed genes under cold stress showing number of genes expressed in common or uniquely in either of the genotypes. *Up* Up-regulated, *Dn* Down-regulated, *Sa* Saral, *Ilc* ILC533.

Based on the inspection of the differentially expressed genes (DEGs), 3710 (1980 up- and 1730 down-regulated) and 3473 (1972 up- and 1501 down-regulated) genes were differentially regulated under cold stress in Saral and ILC533, respectively. According to the comparative transcriptome analysis, 1031 and 647 DEGs were commonly up- and down-regulated in the leaves of the two genotypes. A sum of 949 and 1082 cold-responsive genes in Saral, and 940 and 854 DEGs in ILC533 were exclusively up- and down-regulated, respectively (Fig. 1). Based on different expression patterns of the two studied genotypes, the tolerant and sensitive genotypes somehow utilize diverse mechanisms to respond to cold stress.

### GO classification of DEGs

**Figure 2:**
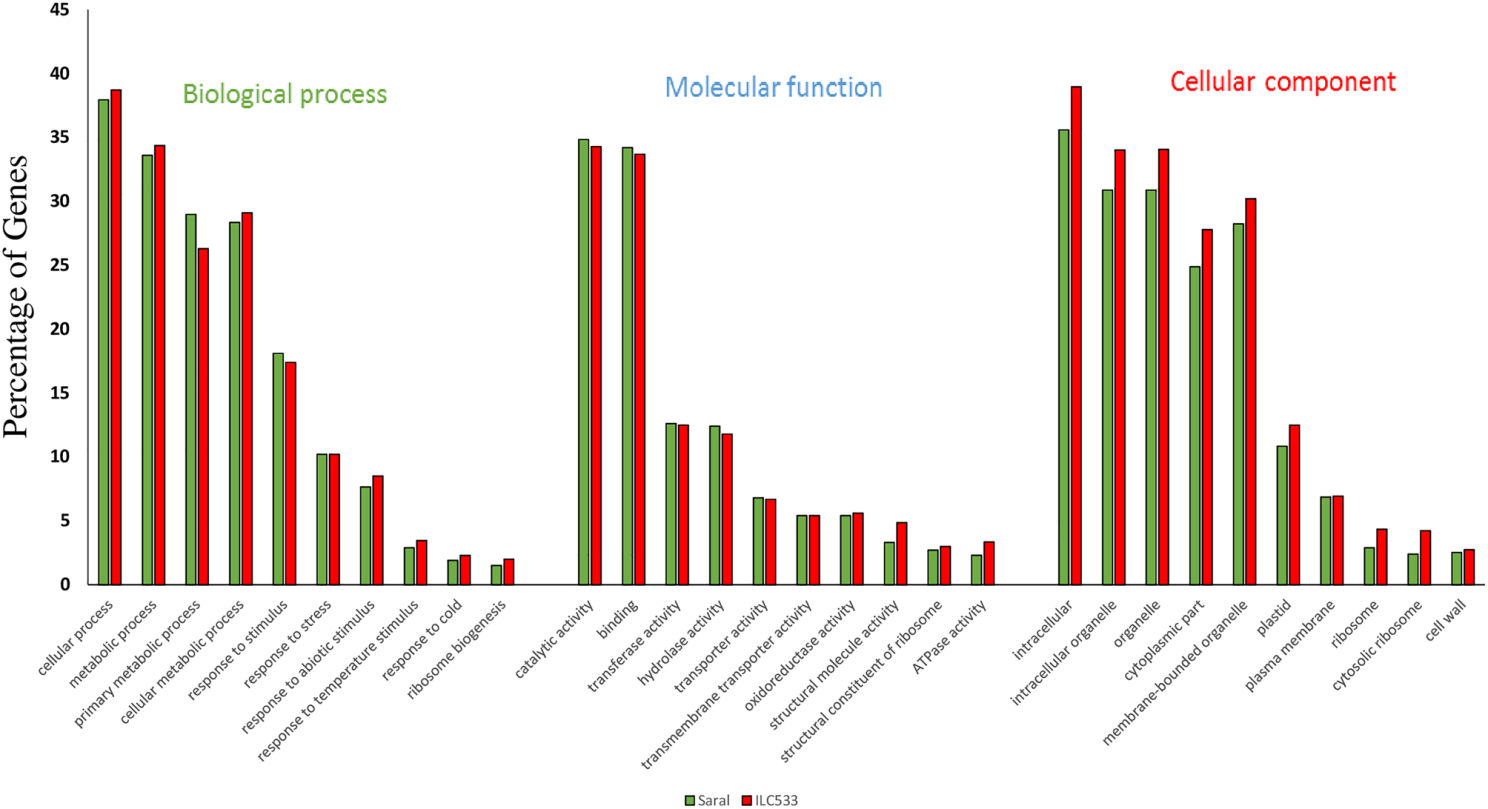
GO categorization of the DEGs in Saral and ILC533 genotypes.

GO analysis of DEGs revealed that 3451 (out of 3710) genes in Saral and 3242 (out of 3473) genes in ILC533 were assigned with GO terms. The GO enrichment analysis of DEGs indicated that some biological processes, including response to stress, abiotic stimulus, temperature, and cold, as well as ribosome biogenesis were enriched in both genotypes (Fig. 2); this is in agreement with prior reports^24–26^. In the molecular function category, catalytic, binding, transferase, hydrolase, transporter, transmembrane transporter, oxidoreductase, and ATPase activity, as well as structural constituent of ribosome were among the highly enriched GO indicators in both genotypes. The most enriched cellular component terms for DEGs of both genotypes were membrane-bounded organelle, plastid, plasma membrane, ribosome, cytosolic ribosome, and cell wall, which are related to plant response to cold stress according to previous studies (Fig. 2). Furthermore, the GO analysis for the genes exclusively up-regulated in the tolerant genotype under cold stress conditions indicated that biological processes including signaling, regulating the response to stress/stimulus, flavonoid, and phenylpropanoid metabolic processes were enriched. On the other hand, GO enrichment analysis of uniquely down-regulated genes under cold stress in ILC533 showed that GO terms such as photosynthetic membrane, photosystem II, chloroplast part, and photosystem processes were enriched.

### KEGG pathway analysis for DEGs

**Figure 3:**
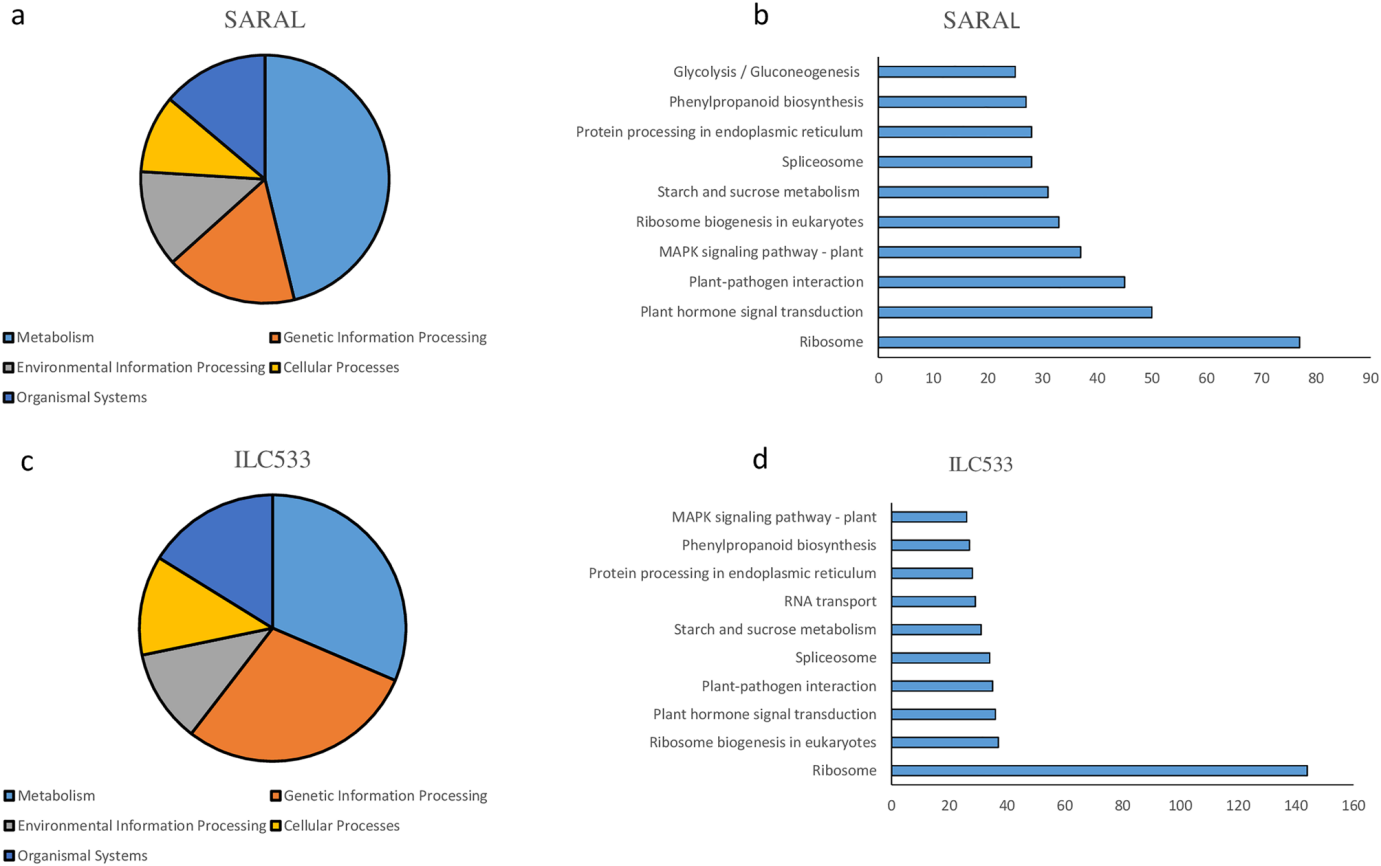
Classification of the DEGs in KEGG pathways: (**a**) and (**c**) Distribution of the DEGs into five main KEGG classes in Saral and ILC535, respectively. (**b**) and (**d**) The top 10 KEGG pathways having the highest number of genes.

To further uncover the biological pathway roles under cold stress in each genotype, the KAAS server was utilized to perform a single-directional BLAST search of DEGs against the KEGG (Kyoto Encyclopedia of Genes and Genomes)^27^ protein database. The results indicated that 1183 DEGs (out of 3710) were categorized in 260 KEGG pathways in Saral (Table S3), and 1200 DEGs (out of 3473) were categorized in 261 KEGG pathways in ILC533 (Table S4). Environmental and genetic information processing, metabolism, organismal systems, and cellular processes were recognized as the main KEGG classes (Fig. 3). In Saral, the top 10 KEGG pathways were ribosome, plant hormone signal transduction, plant-pathogen interaction, MAPK signaling pathway—plant, ribosome biogenesis in eukaryotes, starch and sucrose metabolism, spliceosome, protein processing in the endoplasmic reticulum, phenylpropanoid biosynthesis, and glycolysis/gluconeogenesis, respectively. Ribosome, ribosome biogenesis in eukaryotes, plant hormone signal transduction, plant-pathogen interaction, spliceosome, starch and sucrose metabolism, RNA transport, protein processing in the endoplasmic reticulum, phenylpropanoid biosynthesis, and MAPK signaling pathway – plant, in turn, were recognized as the top 10 KEGG pathways with the most gene numbers in ILC533 (Fig. 3).

### Mapping the DEGs to metabolic pathways using Mapman

The GO and KEGG analysis of the DEGs revealed that cold stress resulted in metabolism changes. The overview of DEGs mapping of each genotype to metabolic pathway indicated that genes engaged in nucleotide metabolism.degredation and mitochondrial electron transports were enriched in both genotypes (Fig. S1 and Table S5). In terms of secondary metabolites, the results showed that the flavonoid metabolism pathway was enriched and the genes engaged in the metabolism of isoprenoids and phenylpropanoids were mapped in both genotypes (Table S5). However, phenylpropanoid and isoprenoid pathways were exclusively enriched in Saral and ILC, respectively, indicating the different responses of the two studied genotypes to cold stress. The overview of DEGs mapping to cellular pathways showed that the stress.abiotic.heat pathway was enriched in the two genotypes. Even though the genes involved in cold stress response and redox.glutaredoxins were mapped in both genotypes, the cold stress response pathway was exclusively enriched in Saral under cold stress (Fig. S2 and Table S5). In addition, the results indicated that the genes coding for miscellaneous enzyme families (misc) and misc.cytochrome P450 were enriched specifically in Saral under cold stress (Table S5). Furthermore, according to the regulation overview, the genes involved in transcription regulation, such as members of MYB-related and Pseudo ARR transcription factor and Constans-like zinc finger families were enriched in both genotypes, while APETALA2/Ethylene-responsive element binding protein and NAC domain transcription factor families were mapped in both genotypes but exclusively enriched in Saral. Furthermore, while signaling.calcium pathway was enriched in both genotypes, more genes were involved in this pathway in the tolerant genotype (Fig. S3 and Table S5).

### Identification of the novel transcripts through mRNA sequencing

The discovery of new genes/transcript isoforms is the core benefit of RNA-seq analysis^28,29^. A total of 60,707 and 61,154 transcripts were recognized in Saral and ILC533, respectively, among which 763 and 787 transcripts were recognized as the novel ones. The average length of the novel transcripts was 1245 bp in Saral and 1311 bp in ILC533, constituting 1.25% and 1.28% of the total transcripts in these two genotypes. Aligning the novel transcripts against the NCBI's nonredundant (nr) protein database using the Blast2GO tool showed that around 68.5% and 68.7% of the transcripts were specified to a putative function in Saral and ILC533, respectively. In addition, 99 (34 up- and 65 down-regulated) and 86 (29 up- and 57 down-regulated) novel DEGs were discovered in Saral and ILC533, respectively. The GO analysis for the novel transcripts in both genotypes indicated that in biological process category, cellular, metabolic, and regulation processes constituted the most highly represented transcripts. In molecular function category, binding, catalytic, transporter and ATP-dependent activities were identified as the dominant terms. Cellular anatomical entity and protein-containing complex terms were assigned to the novel transcripts in the cellular component category (Fig. S4).

### Validation of differential gene expression using qRT-PCR

**Figure 4:**
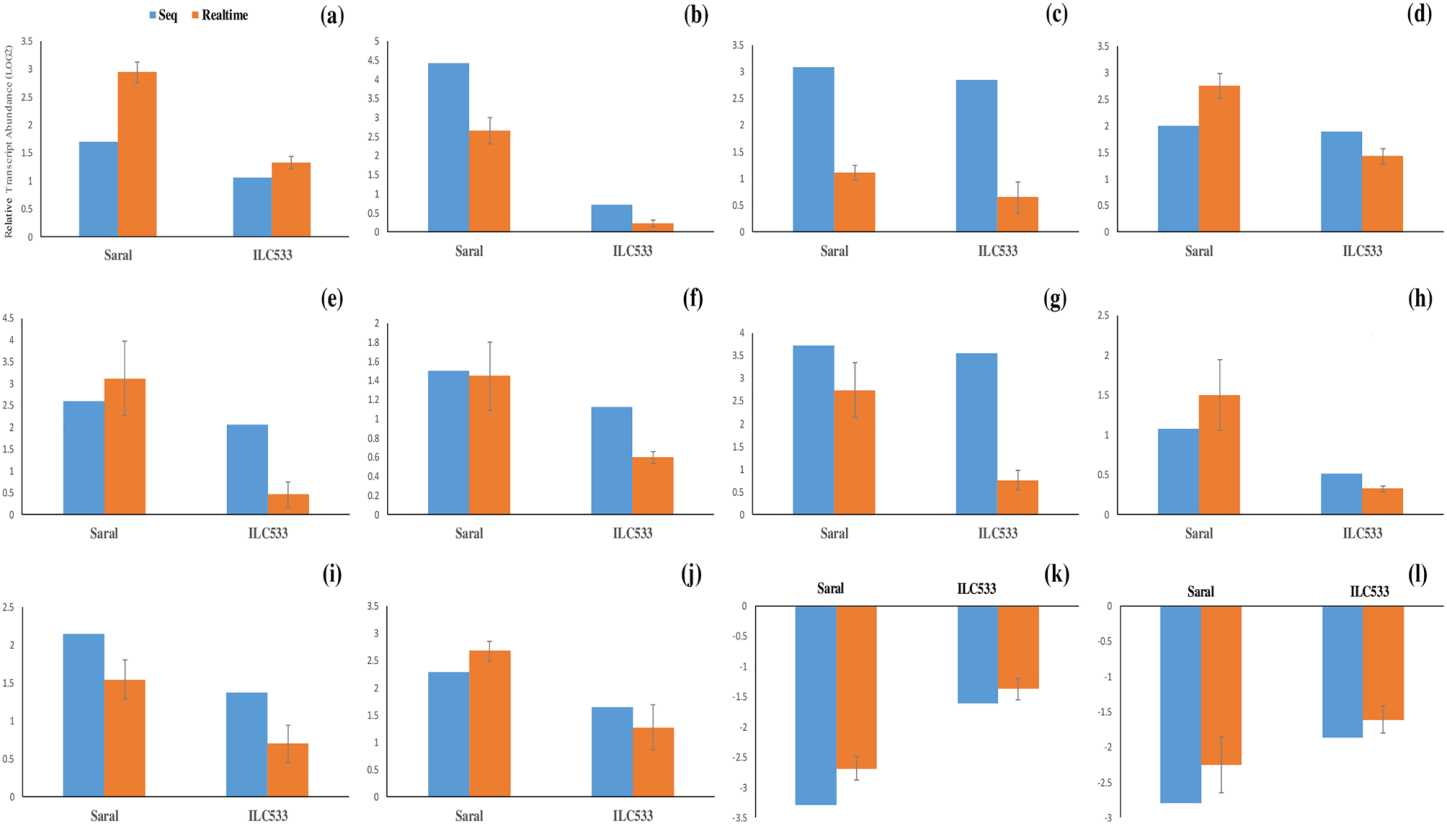
(**a**) *CaMYB4,* (**b**) *Dehydration-responsive element-binding protein 1E-like (CaDREB1E),* (**c**) *CaNAC47,* (**d**) *CaTCP4,* (**e**) *WRKY transcription factor 33 (CaWRKY33),* (**f**) *Calcium-dependent protein kinase4 (CaCDPK4),* (**g**) *Heat stress transcription factor A-3 (CaHSFA3),* (**h**) *Mitogen-activated protein 4-like MKK2 (CaMAPK4),* (**i**) *Dehydrin COR47 (CaCOR47),* (**j**) *Late embryogenesis abundant protein3 (CaLEA3),* (**k**) *Protein phosphatase 2C 6 (CaPP2C6)*. and (**l**) *Polygalacturonase 1 beta-like protein 3 (CaPGL3).*

The expression patterns of 12 cold-responsive genes (Table S1) were inspected by qRT-PCR in the tolerant and susceptible genotypes to confirm the RNA-seq results (Fig. 4). The results of qRT-PCR and RNA sequencing were highly compatible in both genotypes (in Saral; R^2^ = 0.8911 and in ILC533; R^2^ = 0.8079).

## Discussion

Cold is among the key environmental stresses impacting crop production as it limits growth, yield, and quality in crop species^30^. Plants, as sessile organisms, have evolved different physiological, biochemical, and molecular mechanisms to respond to cold. These mechanisms are adjusted by a complex of transcription factors and proteins to raise plant tolerance^31^. Cold tolerance has a quantitative property controlled by several genes. The results of this work provide insights into the expression profiles of cold-responsive genes in two contrasting chickpea genotypes^32^.

According to the GO enrichment analysis of the genes exclusively up-regulated in the cold-tolerant genotype (Saral), the phenylpropanoid metabolic process was significantly enriched under the cold stress condition. Likewise, mapping the DEGs of Saral under cold stress to the secondary metabolites pathway indicated that phenylpropanoids were exclusively enriched. The phenylpropanoid pathway is the main metabolites pathway involved in synthesizing the majority of secondary metabolites, including lignin, lignans, flavonoids, hydroxycinnamic acid amides, phenylpropanoid esters and sporopollenin^33,34^. Accumulation of phenolic compounds, including suberin or lignin, caused the thickness of cell wall to be increased, prohibiting cold stress injury and cell collapse^35,36^. Phenolic biosynthesis enhancement under cold stress is caused by up-regulation of Phenylalanine ammonia-lyase (PAL), cinnamyl alcohol dehydrogenase (CAD), and hydroxycinnamoyl transferase (HCT) expression^37^. In the present research, while significant up-regulation of three genes coding for CAD was observed in Saral, only one gene was significantly induced in ILC533. In addition, the up-regulation of the common *CaCAD* gene in response to cold stress was much higher in Saral compared to ILC533.

Furthermore, the ILC533 DEGs mapping to the secondary metabolites pathway indicated that the isoprenoid pathway was enriched, and most involved genes significantly were down-regulated under cold treatments. Isoprenoids are belonged to a huge and diverse category of volatile organic compounds, which are synthesized from terpenes and have essential functions, including lipids in cell membranes, quinones in the electron transport chain and signal transduction, as well as antioxidants and hormones^38,39^. Isoprene (simplest Isoprenoid) protects plants from different extreme conditions, including drought^40,41^, heat^42–44^ and oxidative stresses^45^. It protects the photosynthetic system through thylakoid membrane stability^46,47^ enhancement and ROS quenching. High destruction resilience of thylakoid membrane in isoprene-emitting plants preserves the better status for molecular diffusion, electron transport, dynamic lumen swelling, and molecular/structural reorganization under heat stress^45^.

GO enrichment analysis of the genes exclusively down-regulated under cold stress in the cold-sensitive genotype (ILC533) indicated that photosystem II, chloroplast part and photosystem process were significantly enriched under cold stress conditions. Photosynthesis, as a principal plant metabolic process, is severely sensitive to cold stress. Low temperature disturbs almost all key components of the photosynthesis apparatus, including Photosystems I and II, photosynthetic pigments, CO2 reduction pathways, and electron transport systems, inhibiting overall photosynthesis^48–50^.

The current research identified many transcription factors (TFs) among the DEGs. TFs have a vital role in cold stress response through transcription adjustment of the downstream genes engaged in plants cold stress tolerance^51^. The APETALA2/Ethylene responsive factor (AP2/ERF), NAC, MYB, TCP4, and Zn-finger have been identified as important TFs engaged in the plant cold stress^16,52,53^ response regulation; such stress-responsive TFs may be significant targets for developing crops with improved cold stress tolerance.

The AP2/ERF is among the large TF families engaged in stress response pathways and developmental processes in plants^54,55^. Several genes from this family were found exclusively cold-responsive in the tolerant genotype (e.g., ethylene-responsive transcription factor RAP2-1-like (LOC101512420), ethylene-responsive transcription factor-like protein (LOC105851094), ethylene-responsive transcription factor TINY-like (LOC101506537), AP2-like ethylene-responsive transcription factor (LOC101498533), dehydration-responsive element-binding protein 1E-like (LOC101505186). C-repeat binding factors (CBFs), recognized as Dehydration responsive element binding proteins (DREBs), are the most popular members of the AP2/ERF family^56,57^. DREBs have a key role in plant stress tolerance and act as the vanguard of plant regulatory networks^57–59^. They can activate the expression of COR (cold-related), RD (Responsive to Dehydration), LTI (Low-temperature Induced), and other cold-regulated genes^16,60^. The CBFs’ overexpression enhances cold tolerance by increasing antioxidant enzymes such as catalase (CAT), peroxidase (POD), ascorbate peroxidase (APX), superoxide dismutase (SOD), as well as proline and reducing MDA, H_2_O_2_, and O^-2^ content^61–63^. The overexpression of the *BpERF13* gene in white birch significantly improves cold tolerance via up-regulation of CBF genes and decrease in reactive oxygen species accumulation^64^.

One of the recognized candidate genes in the present study was *dehydration-responsive element-binding protein 1E-like* (*CaDREB1E*, LOC101505186), which was highly up-regulated in the tolerant genotype in response to cold stress; however, its induction was not significant in the sensitive line (Fig. 4b). Previous studies also have indicated that the overexpression of *AtDREB1* enhances freezing tolerance in transgenic Arabidopsis^65^, potato^66^, and tobacco^67^. Overexpression of the DREB/CBF genes results in biochemical variations related to cold tolerance^68,69^. The *OsDREB1A*, *OsDREB1B,* and *OsDREB1C* interaction with the GCC box increase the cold tolerance of the rice plants^70^. Chen et al. stated that the overexpression of rice *DREB1E* enhanced plant survival rate under water-deficient conditions^71^.

Based on the results of the current study, *CaMYB4* (LOC101508022) was significantly up-regulated in both genotypes but higher increase was observed in the susceptible line (Fig. 4a). The MYB superfamily, one of the most abundant classes of TFs in plants, holds a substantial quota in cold stress response^72^. The MYBs' role in cold stress response has been further recognized by functional studies using overexpression and knock-out systems^73^. Transgenic Arabidopsis plants with overexpression of *Osmyb4* have shown improved cold stress tolerance^74^. The overexpression of *Osmyb4* in Arabidopsis leads to multiple metabolic changes (free amino acids) commonly observed in plants during cold acclimation^75,76^. Furthermore, an increase in soluble sugars, leaf chlorophyll content, and superoxide dismutase activity, as well as a reduction in malondialdehyde (MDA) content, under chilling stress have been reported in *LcMYB*4-overexpressing Arabidopsis. Indeed, *LcMYB4* overexpression enhances soluble sugar content and cold-inducible gene expression and attenuates oxidative and membrane damage, resulting in cold tolerance^77^.

Based on the results, up-regulation of *CaNAC47* (XM_004503844) was observed in both genotypes, while its induction was more in the tolerant genotype (Fig. 4c). NAC transcription factors have a fundamental role in responses to stresses in plants^78^. The role of NACs has been considered and recognized in different plants, including Arabidopsis^79^, rice^80^, peppers^81^, and *Medicago truncatula*^82^, under cold stress conditions. ABA hypersensitivity and improved tolerance to salt, drought, and freezing have been demonstrated in transgenic Arabidopsis plants with overexpression of *TaNAC47.* In addition, increased soluble sugars and proline contents have been reported in *TaNAC47* overexpressing plants after exposure to drought and cold treatments^79^.

In the present study, cold stress led to up-regulation of *CaTCP4* (LOC101506032) in both cultivars; however, more increase was observed in Saral genotype (Fig. 4d). TCP transcription factors are a plant-specific category with fundamental roles during the development of plants and their responses to cold stress^83–85^. The overexpression of *MeTCP4* of Cassava (*Manihot esculenta*) in Arabidopsis led to enhanced cold tolerance by increasing proline content and reducing cell membrane damage. Furthermore, much higher expression of ROS-scavenging-related genes such as *GSTF7*, *GSTU12*, and *FRO3* was detected in *MeTCP4* overexpressing plants as compared with the wild type under cold stress conditions^86^. Glutathione S-transferases (GSTs), recognized as ubiquitous and multifunctional proteins, inhibit oxidative damage^87^. They are involved in cold, drought, salt, and oxidative stress tolerance in Arabidopsis^88^. The up-regulation of *GSTs* (LOC101508652, LOC113783892) was also observed in the tolerant genotype in the current investigation.

Based on the present research results, *CaWRKY33* (LOC101509113) was substantially up-regulated in the tolerant genotype in response to cold stress, while its induction was not statistically significant in the sensitive cultivar(Fig. 4e). The WRKY TF family is among the important transcription factor families in higher plants^89,90^. WRKY TFs are recognized as essential regulators in various physiological and developmental processes^89^ as well as abiotic stress responses, including cold stress^91,92^. The overexpression of *CsWRKY46* from cucumber in Arabidopsis resulted in higher seedling survival rates under freezing stress compared to the wild type. This overexpression enhanced cold tolerance in Arabidopsis via expression regulation of stress-induced genes such as *RD29A* and *COR47* in the ABA-dependent manner. The up-regulation of *COR47* (LOC101512214) and a chloroplastic early *responsive to dehydration* (LOC101495575) were also observed in present study.

Furthermore, the expression of a regulatory gene called probable *protein phosphatase2C6* (*CaPP2C6*, LOC101510725), which negatively affects stress tolerance, decreased under cold stress in both genotypes. However, its down-regulation was greater in Saral compared to ILC533 under cold stress (Fig. 4k). Type 2 C protein phosphatases (PP2Cs), the main class of plant protein phosphatases, have converse functions in stress signaling pathways in various plant species^93–95^. The negative regulatory functions for *ZmPP2C-A10* have been demonstrated in maize and Arabidopsis under drought stress^96,97^. Moreover, the suppression of *AtPP2CA* expression caused cold acclimation and enhanced freezing tolerance in Arabidopsis^98^. Certain PP2C genes are engaged in the ABA signaling cascade regulation by changing the kinase activity, MAPK or SnRK, under abiotic stress conditions^97^.

Signal perception and transduction, as well as the expression of stress-responsive genes, are the basic ingredients in stress responses^99^. In the current research, cold stress led to significant up-regulation of *calcium-dependent protein kinase 4* (*CaCDPK4*, LOC101492192) in the tolerant genotype; however, its induction was not significant in the susceptible line (Fig. 4f). *CDPK4* is a calcium-dependent protein kinase (CDPK) gene family member. Several CDPK genes are transcriptionally altered by cold stress^100^. The overexpression of *PeCPK1*0 resulted in more proline accumulation and caused freezing tolerance of transgenic Arabidopsis^101^.

In the present research, *CaHSFA3* (XM_004497545) was up-regulated in both genotypes under cold conditions, more in the tolerant genotype (Fig. 4g). Plant Heat-Shock Factors (HSFs) coded by extensive gene families are divergent from expression, function, and structure points of view. HSFs are members of complex signaling systems that regulate responses to different abiotic stresses, including cold, high temperatures, salinity, drought and oxidative stress^102^. They are engaged in increasing the expression of HSPs, such as HSP90s, HSP70s, and some small HSPs^103,104^. Genes encoding HSP70/90 and HsfA3/A8 are not only regulated by temperature stress, but also interact with chlorophyll synthesis and peroxide scavenging processes under cold stress^105^. The overexpression of *TaHSF3* seriously increased resilience to freezing and heat stresses by inducing HSP70s in transgenic Arabidopsis plants^106^. Additionally, *OsHsfA3* is particularly induced in both the shoot and root tissues of rice under cold stress^107^.

The present study showed that mitogen-activated protein kinase 4-like (*CaMKK2*, XM_004492727) was up-regulated in the tolerant genotype under cold conditions, whereas its induction was not significant in the sensitive line (Fig. 4h). Mitogen-activated protein kinase (MAPK) cascades are popular signal transduction pathways in all eukaryotes with fundamental roles^108,109^. The MAPK cascade controls plant tolerance to temperature stresses by phosphorylating downstream targets to directly alter related gene expression and cellular metabolism (enhancing compatible solutes and antioxidative enzyme activities)^110,111^. Transgenic tobacco plants overexpressing *SlMPK3* from tomato exhibited enhanced antioxidant activity, raised proline and soluble sugars content, and improved cold tolerance^112^. MEKK1-MKK2-MPK4/6 pathway positively controls cold response and freezing tolerance in Arabidopsis^113^. Under low temperatures, MEKK1 is activated and subsequently phosphorylates MKK2^114^. Phosphorylated MKK2 activates MPK4 and MPK6 involved in regulating downstream components to cope with low-temperature stress conditions^113^. The mkk2 mutant plants exhibited enhanced susceptibility to freezing, while transgenic plants that expressed a constitutively active form of MKK2 showed enhanced freezing tolerance by increasing the CBF genes' expression^113^.

The present study indicated a greater down-regulation for the gene coding polygalacturonase 1 beta-like protein 3 (*CaPGL3*, LOC101490440) in the tolerant genotype as compared with the sensitive genotype (Fig. 4l). Polygalacturonases (PGs) are enzymes necessary for the degradation of cell wall pectin^115^. It was shown that the overexpression of *OsBURP16*, a member of the PG1β-like subfamily, increased sensibility to cold, drought and salinity stresses compared to controls in rice. The *OsBURP16* overexpression led to pectin degradation, affecting the integrity of cell wall and transpiration rate, and caused abiotic stress tolerance to be reduced^116^. Instead, it has been shown that cold acclimation increases cell wall pectin content and enhances freezing tolerance^117^.

Based on the obtained results, cold stress led to the up-regulation of *CaLEA3* (LOC101508885) in both genotypes, mostly in the tolerant genotype (Fig. 4j). Late embryogenesis abundant (LEA) proteins, recognized as small molecule-specific peptides, are created in the late step of seed development, helping plants deal with diverse abiotic stresses^118^. Members of the LEA gene family are regulated and expressed under various stress conditions. Different studies show the involvement of LEA proteins in cold stress tolerance in different plants. The overexpression of the wheat LEA gene (*WCOR410*) increased cold tolerance in transgenic strawberry plants^118^. Salt and drought stress tolerance simultaneously increased in wheat and rice plants overexpressing barley LEA (*HVA1*) gene. The *ZmLEA3* overexpression in tobacco resulted in increased cold tolerance^119^.

Another candidate gene identified in the current study is dehydrin (*CaCOR47*, LOC101512214), playing a role in the cold tolerance of chickpeas. *CaCOR47* was up-regulated in both genotypes under cold stress; however, more rise in its expression was observed in the tolerant genotype under cold stress (Fig. 4i). COR47 is a member of the group II LEA proteins^120,121^. COR (cold-responsive) genes are quickly induced by cold stress during cold acclimation^122^. They are generally up-regulated by numerous abiotic stresses through binding of CBFs to the related *cis*-elements located in their promoters. Simultaneous overexpression of *COR47* and *RAB18* genes increased freezing tolerance in Arabidopsis, which could be partly due to their protective effect on membranes^123^.

## Conclusions

**Figure 5:**
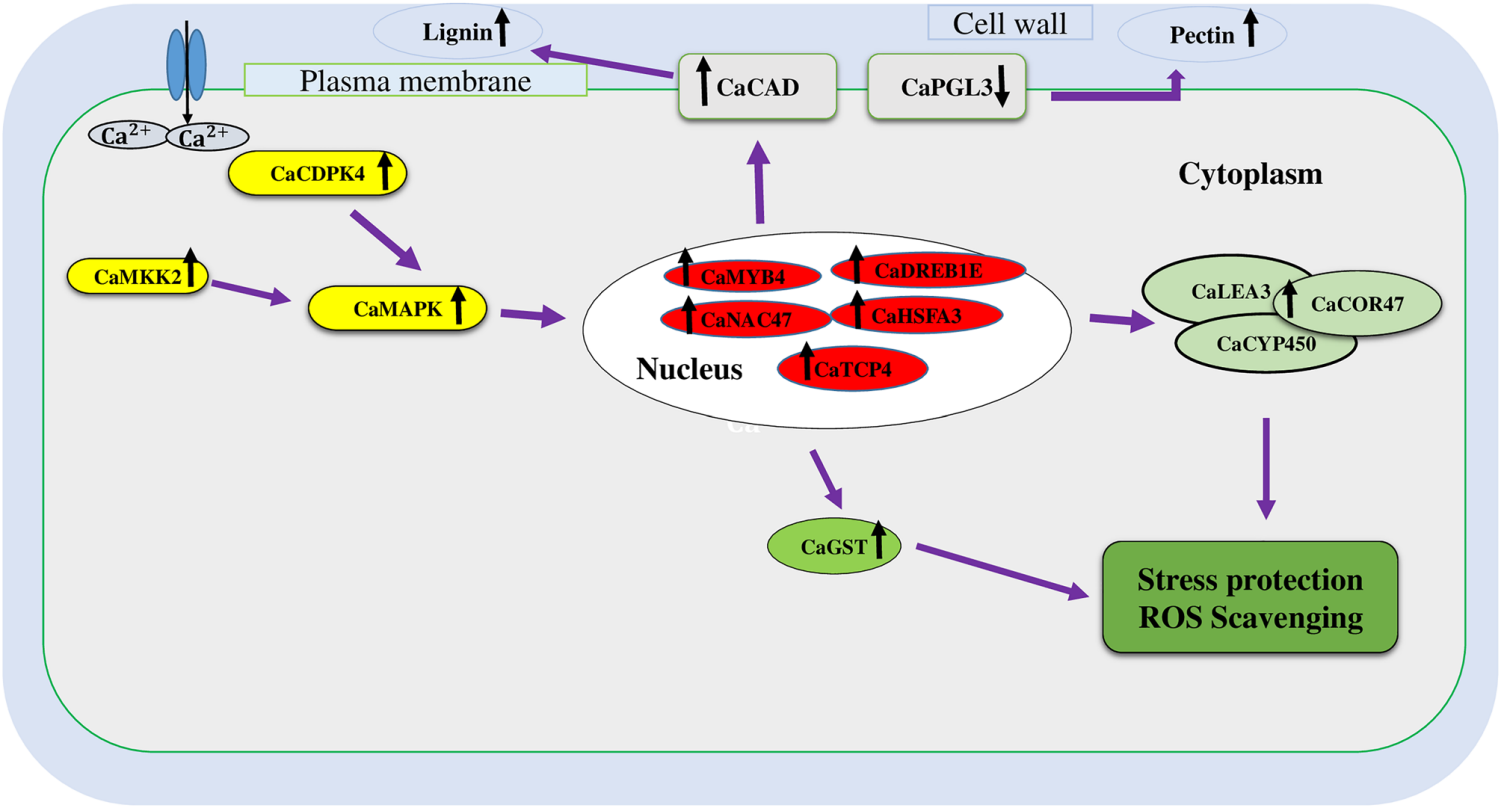
Proposed model for cold tolerance in a tolerant cultivar of chickpea, saral. Yellow and red colors were utilized to depict signaling-associated genes and transcription factors, respectively. White and green colors were used to exhibit genes involved in cell wall modifications and stress-protective and ROS scavenging genes, respectively.

According to the comparative analysis of transcriptional responses to cold stress in Saral (as a Kabuli tolerant genotype) and ILC533 (the sensitive line), the former employed more efficient mechanisms to enhance cold tolerance (Fig. 5), including 1) Smart regulation of signaling genes (e.g., *CaCDPK4, CaMKK2* and *CaHSFA3*) and TFs (e.g., *CaDREB1E, CaMYB4*, *CaNAC47* and *CaTCP4*), 2) Up-regulation of several stress-protective proteins (e.g. *CaLEA3*, *CaCOR47*) and ROS-scavenging genes (GSTs), 3) Preserving crucial plant metabolism processes, such as photosynthesis, 4) Enrichment of the phenylpropanoid metabolic process (e.g., *CaCAD*), which are involved in synthesizing secondary metabolites including lignin, leading to thickening the cell wall and prohibiting cold stress injury, 5) Down-regulation of cell wall pectin degradating enzyme (*CaPGL3*). These results would improve the understanding of the genetics underlying cold stress tolerance, which could eventually benefit the enhancement of cold tolerance across chickpea genotypes.

## Material and methods

### Plant growth and cold stress treatment

Two Kabuli chickpea genotypes, Saral (cold tolerant) and ILC533 (cold susceptible), were included in this study. The seeds were obtained from the Dryland Agricultural Research Institute of Iran. They were sterilized for 10 min in Sodium hypochlorite (1%) (NaClO), washed with distilled water, and placed on moistened filter papers. After three days, the uniform germinated seeds were transferred to pots filled with soil composed of a mixture of field soil, sand, and peat moss in a volume ratio of 2:1:1. The pots were placed in a phytotron at 20 ± 3 °C temperature, 16/8 (day/night) photoperiod, and relative humidity of 75%. At the 4–5 leaf stage, one-half of the pots were exposed to 4 °C, and the rest remained at 20 ± 3 °C. After 12 hs, sampling was done from plants grown under both conditions. The collected leaf samples were put in liquid nitrogen immediately and kept at − 80 °C in a freezer.

### RNA extraction and mRNA sequencing

The total RNA was extracted from three biological replicates of both control and cold-treated samples using RNeasy Plant Mini Kit (Qiagen) based on the manufacturer's guidelines. Integrity, quantity, and quality of extracted RNA were evaluated by agarose gel electrophoresis, nanodrop, and Agilent Bioanalyzer 2100 system (Agilent Technologies Co. Ltd., Beijing, China). The cDNA libraries were constructed from two biological replicates, and sequencing by Illumina Hiseq 2500 platform (Novogene Bioinformatic Institute, Beijing, China) resulted in generating 150 bp paired-end reads. The filtering process was done to remove adapters containing reads, reads with N > 10% and containing low quality (Qscore ≤ 5) base of more than 50% of the total bases.

### Quality control and RNA-seq data

The raw FastQ data quality was evaluated using the FastQC toolkit. The high-quality reads were mapped against the chickpea reference genome (https://www.ncbi.nlm.nih.gov/genome/2992) utilizing TopHat. Cufflinks created a reference annotation-based transcript (RABT) assembly using the resulting alignment reads from each sample and the genome GFF. The individual assemblies were merged to create the whole assembly applying Cuffmerge with default parameters. Furthermore, Cuffmerge was applied to identify novel transcripts^124^. Cuffdiff, in the Cufflinks package, was used to identify differentially expressed genes (DEGs). Log2 fold change ≥ 1 or ≤  − 1 and Q-value ≤ 0.01 were utilized as thresholds to recognize significant DEGs. DIAMOND124 was utilized to align the DEGs against the NCBI nonredundant protein database via BlastX with a threshold e-value of 1e^-3125^.

### Functional annotation and pathway analysis of DEGs

For each genotype, GO terms were assigned to DEGs using AgriGO at an FDR cut-off of 0.05. The involvement of DEGs in KEGG pathways was recognized by utilizing the Online KEGG Automatic Annotation Server (KAAS) (https://www.genome.jp/kegg/kaas/). In addition, for pathway analysis of DEGs, Mapman (version 3.5.1; http://mapman.gabipd.org/web/guest) with a *p*-value threshold ≤ 0.05 was applied. Mapping DEGs on Arabidopsis pathway genes resulted in identifying genes engaged in particular pathways^126^.

### Real-time PCR analysis

In order to validate the RNA-seq results, Real-Time PCR was employed. Twelve genes were chosen from the panel of cold-responsive genes obtained in the RNA-seq experiment. Oligo 7.0 (ver. 5.0; National Bioscience Inc., Plymouth, USA) was utilized to design gene-specific primers. Primers designed for the chosen genes are itemized in Table S1. IScript™ cDNA synthesis kit (Sina clon) was used for cDNA synthesis. LightCycler^®^ 96 Real-Time PCR System (Roche Life Science, Germany) and SYBR Premix Mix Green High Rox (AMPLIQON, Denmark) were used to perform qRT-PCR on three biological replicates of control and cold-treated leaf samples. *GAPDH* was utilized as a proper internal control gene to normalize gene expression value^13,127^. The relative transcript levels of the candidate genes were obtained from cycle thresholds applying the 2^−ΔΔCt^ process^22,23^. All methods were performed in accordance with relevant institutional (ABRII), national, and international guidelines and legislations.

## Supplementary Information

Supplementary Figures.

Supplementary Table S1.

Supplementary Table S2.

Supplementary Table S3.

Supplementary Table S4.

Supplementary Table S5.

## Data Availability

All the sequencing reads generated from Illumina HiSeq 2500 RNA-Seq are available in NCBI SRA: SRR22402557, SRR22403404, SRR22403635, SRR22403923, SRR22404408, SRR22404851, SRR22404839, SRR22405780 (https://submit.ncbi.nlm.nih.gov/subs/sra/). All other datasets supporting this study are included in the article and its supplementary material.
