# Supplementary material for: Genome-wide transcriptional profiling provides clues to molecular mechanisms underlying cold tolerance in chickpea
Source: Sci Rep. 2023 Apr 18;13:6279. doi: 10.1038/s41598-023-33398-3 (PMC10113226; doi:10.1038/s41598-023-33398-3)
Supplement: Supplementary file 1 — Supplementary Figures. [file 41598_2023_33398_MOESM1_ESM.pdf]

(a)

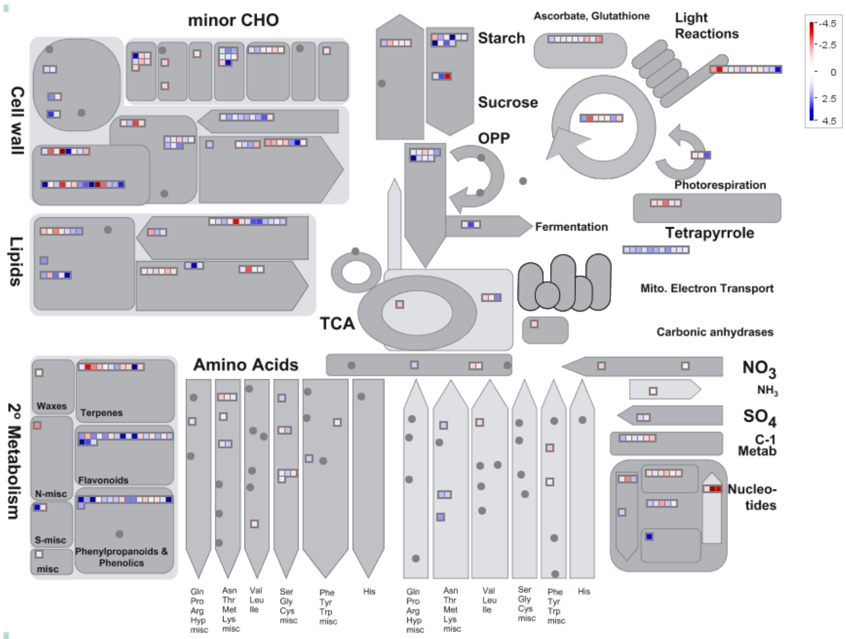

(b)

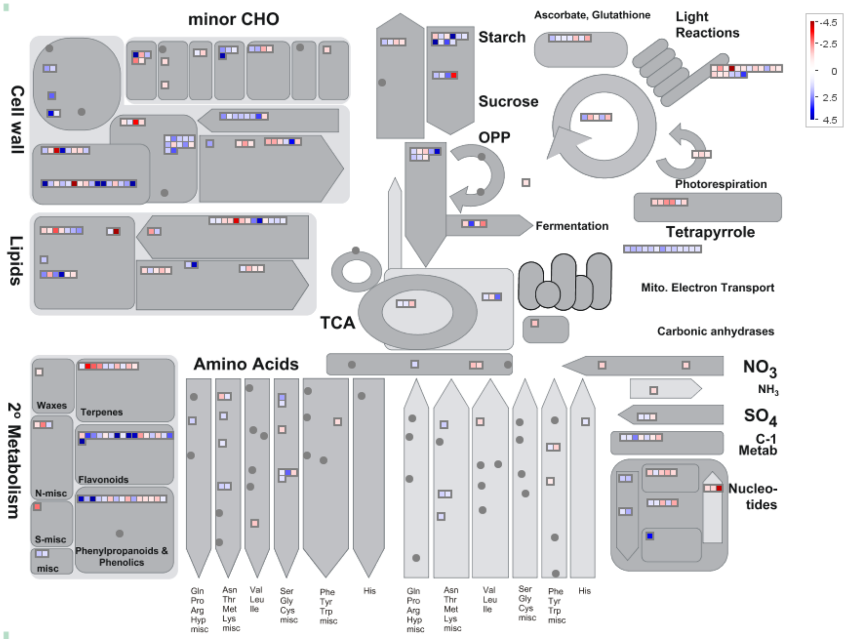

Fig S1. Metabolic pathway overview of differentially expressed genes in *Cicer arientinum* under cold stress in Saral (a) and ILC (b) using Mapman. blue,up-regulated genes and red,down-regulated genes.

(a)

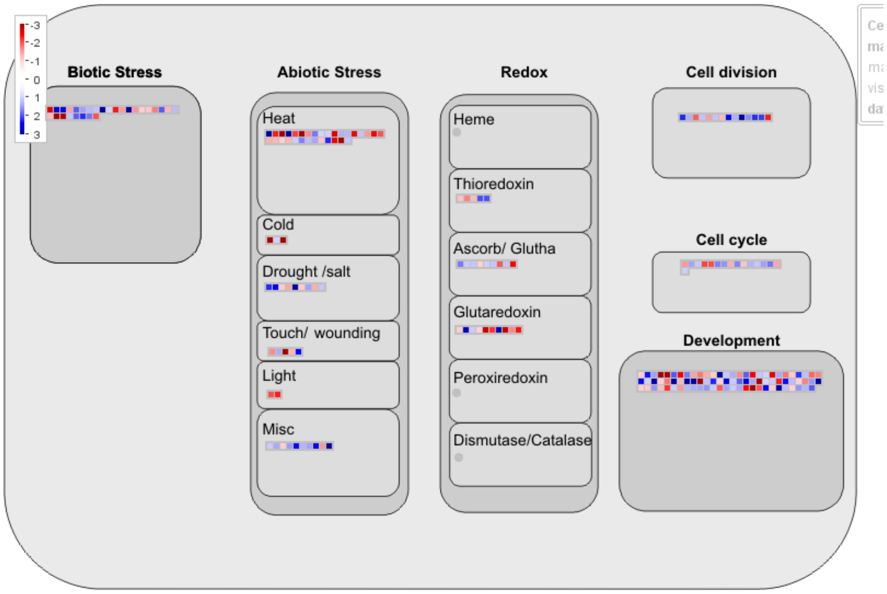

(b)

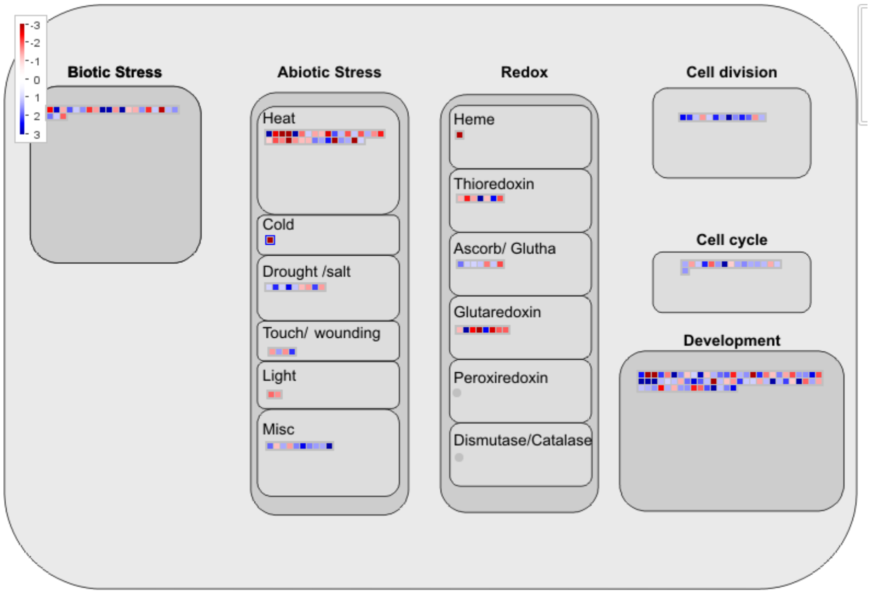

Fig S2. Cellular pathway overview of differentially expressed genes in *Cicer arientinum* under cold stress in Saral (a) and ILC (b) using Mapman. blue,up-regulated genes and red,down-regulated genes.

(a)

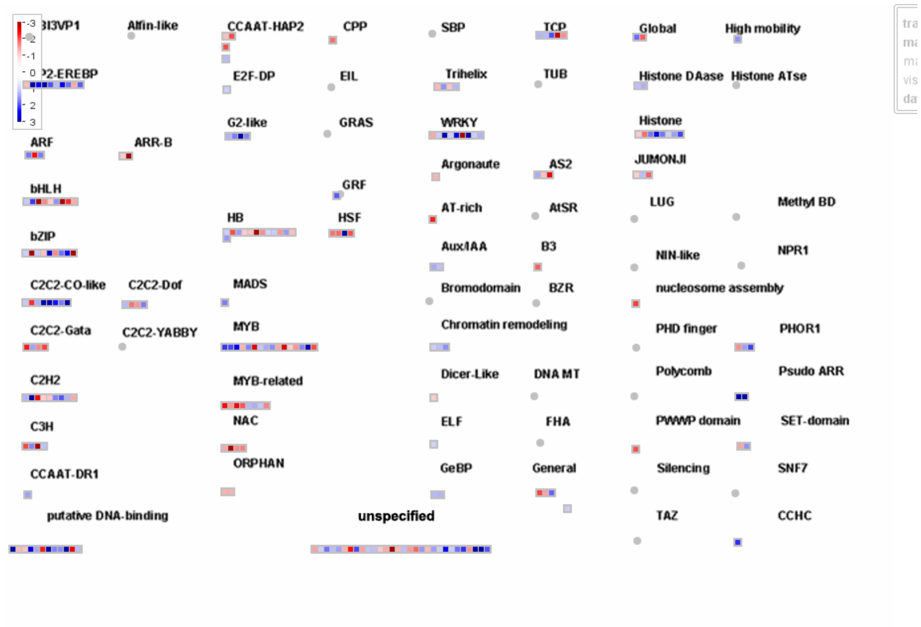

(b)

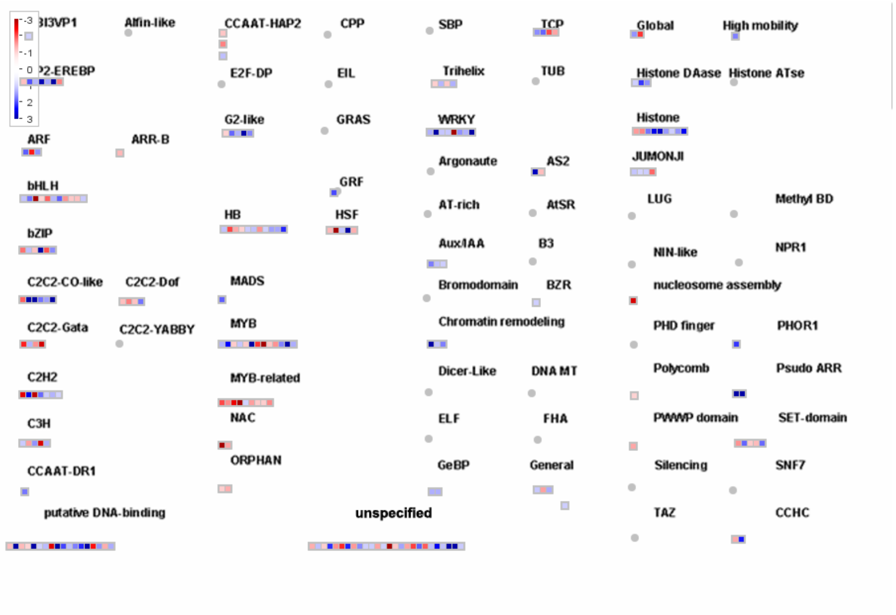

Fig S3. Transcription overview of differentially expressed genes in *Cicer arientinum* under cold stress in Saral (a) and ILC (b) using Mapman. blue,up-regulated genes and red,down-regulated genes.

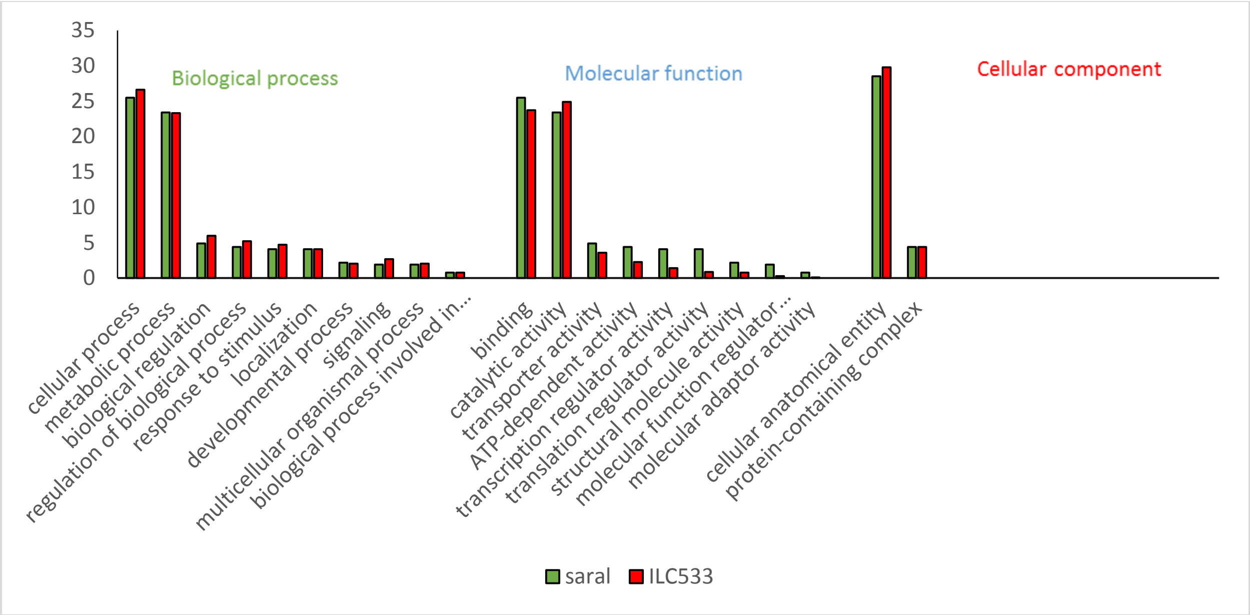

Fig S4. GO classification of the novel DEGs under cold stress.
