# Supplementary material for: Genome-wide transcriptional profiling provides clues to molecular mechanisms underlying cold tolerance in chickpea
Source: Sci Rep. 2023 Apr 18;13:6279. doi: 10.1038/s41598-023-33398-3 (PMC10113226; doi:10.1038/s41598-023-33398-3)
Supplement: Supplementary file 3 — Supplementary Table S2. [file 41598_2023_33398_MOESM3_ESM.pdf]

Table S2. Summary of the sequencing results

Sample name	Raw read(paired end)	Clean reads(pair end)	Q20%	Q30%
**Saral control (rep1)**	24,227,162	24,024,561	95.71	89.11
**Saral control (rep2)**	25,770,158	25,530,558	95.94	89.59
**Saral stress (rep1)**	21,683,054	21,445,932	95.68	88.98
**Saral stress (rep2)**	26,853,443	26,639,044	96.01	89.71
**ILC533 control (rep1)**	24,602,737	24,361,775	95.49	88.70
**ILC533 control (rep2)**	24,035,060	23,814,758	95.96	89.62
**ILC533 stress (rep1)**	27070044	26773138	96.28	90.22
**ILC533 stress (rep2)**	26,611,955	26,412,847	96.02	89.75
**Total**	200853613	199002613	≥95.49	≥88.70
